# Evaluation of the Effect of Mega MSM on Improving Joint Function in Populations Experiencing Joint Degeneration

**Published:** 2015-06

**Authors:** Gang Xu, Tian Zhou, Yaqin Gu, Qinping Wang, Mina Shariff, Pingping Gu, Tuong Nguyen, Rong Shi, Jianyu Rao

**Affiliations:** 1 School of Public Health, Shanghai Jiao Tong University, China;; 2 Beijing University of Chinese Medicine;; 3 Tangqiao Community Health Service Center, Pudong New District, China;; 4 Department of Research, DRM Resources, 1683 Sunflower Avenue, Costa Mesa, CA 92626, USA;; 5 Department of Pathology and Laboratory Medicine, in UCLA Medical Center, University of California, Los Angeles, CA, USA

**Keywords:** Joint degeneration, joint pain, joint stiffness, joint swelling, Mega MSM, quality of life

## Abstract

Joint degeneration has become a commonplace problem in aging populations. The main clinical manifestations include joint pain, joint stiffness and joint swelling with functional disorder. Mega MSM is a nutritional supplement that may provide potential relief for joint problems associated with joint degeneration. The current experiment performed was a 12-week, randomized, double-blind, controlled study conducted on populations in China experiencing joint degeneration. The objective of the study was to determine whether the daily use of Mega MSM capsules could improve joint function, relieve symptoms of joint degeneration and improve the quality of life in aging populations. A total of 100 male and female participants over 50 years old who had at least one of the related symptoms of joint degeneration (joint pain, joint stiffness, joint swelling, difficulty walking, difficulty getting up from bed and difficulty going down stairs) were recruited and their symptoms of joint degeneration before and after the intervention were recorded. In this study, Mega MSM shows positive effects in improving joint function, relieving symptoms associated with joint degeneration and improving the quality of life in aging populations.

## INTRODUCTION

Joint degeneration has become a commonplace problem in aging populations (1). The main clinical manifestations includes joint pain, joint stiffness and joint swelling with functional disorder (2).

Joint concerns have become very common among the elderly populations, and are a major cause of impaired activities of the elderly (3). The prevalence rates of joint concerns among men and women aged above 60 years were, respectively, 21.5% and 42.8% in Beijing (4), and 24.0% and 41.6% in Pudong New District, Shanghai (5) where the study was held.

Preventing and controlling the symptoms of joint degeneration effectively can not only improve the life quality of the elderly and reduce the knee replacement rate for joint health patients (6), but can also reduce the economic burden of the whole society (7).

Mega MSM is a nutritional supplement containing ingredients such as methylsulfonylmethane, vitamin C and collagen that help promote healthy joint cartilage synthesis to aid joint strength, comfort, mobility and flexibility. It is theorized that by supporting joint cartilage formation, Mega MSM might provide potential relief of joint problems associated with joint degeneration. The current experiment performed was a randomized, double-blind, controlled study conducted on populations in China suffering from joint degeneration. A total of 100 eligible participants were recruited and randomly allocated into two groups to receive either Mega MSM or placebo capsules. All participants were followed up for 12 weeks. The changes of related symptoms (including joint pain, joint stiffness, joint swelling, difficulty walking, difficulty getting up from bed and difficulty going down stairs) and self-reported quality of life were recorded to assess the efficacy of Mega MSM on improving joint flexibility and elasticity. The objective of the study was to determine whether the daily use of Mega MSM capsules could relieve symptoms of joint degeneration and improve joint function in aging populations.

## METHODS

### Participants

A total of 100 participants were recruited from the outpatient department of the community health service center of Tangqiao in Shanghai, China, from September 29, 2013, to November 26, 2013. All potential participants were assessed by physical examinations.

All of the participants were men and women over 50 years old who had at least one of the related symptoms of joint degeneration (joint pain, joint stiffness, joint swelling, difficulty walking, difficulty getting up from bed and difficulty going down stairs). The study excluded individuals who had cancer, coagulation dysfunction history, gallstones, stomach ulcers and those who had used bromelain, antibiotics (such as amoxicillin or tetracycline) or anti-platelet drugs within 30 days.

All participants provided written, informed consent for the study.

### Technical Information

All of the 100 eligible participants were randomly assigned into the “Mega MSM group” or the “placebo group” with the ratio of 1:1. Each participant received a container marked with different-colored labels (which was blinded for both study subjects and researchers) and detailed instructions of administrating the capsules. The unmasking was done at the end of the intervention. All patients were required to complete a questionnaire which recorded their date of birth, gender, ethnicity, medical history, family history, smoking and drinking habits, current medication and symptoms of joint degeneration, as well as a 36-item Short-Form Health Survey (SF-36 scale).

The Mega MSM and placebo capsules were both manufactured by Robinson Pharma, Inc (Costa Mesa, California, USA). Participants in the Mega MSM group took two Mega MSM capsules orally, twice daily (after breakfast and lunch). Participants in the control group (placebo group) also took two placebo capsules orally, twice daily (after breakfast and lunch). The active components of the Mega MSM capsules comprised of vitamin C, methylsulfonylmethane, collagen (from chicken), neem (*Azadirachta indica*) extract and corydalis (*Corydalis yanhusuo*) extract. The main ingredient of the placebo capsules was flour.

The SF-36 scale is a widely recognized health-related quality of life (HRQoL) assessment tool used in Europe and America to evaluate both physical and mental health (3). SF-36 includes eight dimensions, including physiological functioning (PF), role-physical (RP), bodily pain (BP), general health (GH), vitality (VT), social functioning (SF), role-emotional (RE) and mental health (MH). In addition, there is an item indicator—health transition (HT) in the SF-36 scale—to assess the changes in health in the past year.

The total intervention period lasted for 12 weeks. All participants were followed up once per month. Capsules were dispensed with follow-ups. All follow-ups for both the Mega MSM group and placebo group were completed on February 26, 2014. A total of 99 cases completed the last follow-up and final survey, (49 cases in the Mega MSM group, 50 cases in the placebo group). One volunteer in the Mega MSM group dropped out.

### Statistical analysis

EpiData 3.02 software was used for the establishment of the database. SPSS 20.0 software was used for statistical analysis. Variables were compared between the two groups by applying Student’s t-test for quantitative variables and Chi-square test for categorized variables. The alpha level chosen was 0.05. Analyses were conducted using intent-to-treat (ITT) analysis. All *p*-values reported were 2-sided.

## RESULTS

### Baseline data

At the baseline and at the end of the intervention period, physicians evaluated the symptoms for every participant through physical examinations, assigning scores ranging from 1 to 5, with 1 indicating the absence of a given symptom and 5 indicating a severe degree of the symptom. The age, gender, smoking history, alcohol intake history, medical history and medication history of all participants were also collected at the baseline. Along with the follow-ups, self-reported side effects and adherences were recorded.

The baseline characteristics between the two groups were comparable on age, gender, smoking history, alcohol intake history and medication history. In the Mega MSM group there were 14 males (28.6%) and 35 females (71.4%), while in the placebo group there were 9 males (18.0%) and 41 females (82.0%). The average age of participants was 68.00 ± 8.19 years in the Mega MSM group and 68.45 ± 9.98 years in the placebo group. The drinking rate in the Mega MSM group and the placebo group were 6.1% and 10.0%, respectively. The smoking rate was 6.1% in the Mega MSM group and 2.0% in the placebo group.

Between the two groups, there were no significant differences in total SF-36 scores, scores of each dimension and physical composite scores (PCS), and mental composite scores (MCS). The only exception were the scores for mental health (MH) (Table 1).

**Table 1:** SF-36 scores at baseline

Dimension	SF-36 ( X ± S )		*t*	*P*
	Mega MSM (n=49)	Placebo (n=50)		

PF	54.59 ± 23.71	61.00 ± 18.98	-1.486	0.141
RP	32.65 ± 45.13	49.50 ± 49.36	-1.773*	0.079
BP	60.61 ± 13.76	64.40 ± 14.02	-1.357	0.178
GH	58.16 ± 23.38	56.40 ± 18.55	0.416	0.678
VT	72.96 ± 18.28	69.90 ± 15.89	0.889	0.376
SF	67.09 ± 22.63	72.00 ± 18.48	-1.183	0.240
RE	97.28 ± 14.96	89.33 ± 28.92	1.722^a^	0.089
MH	83.76 ± 8.92	74.00 ± 15.36	3.842^a^	0.000
HT	43.88 ± 13.04	44.00 ± 11.91	-0.049	0.961
PCS	206.02 ± 88.13	231.30 ± 86.05	-1.444	0.152
MCS	321.09 ± 46.71	305.31 ± 51.52	1.595	0.114
Total score	570.98 ± 132.73	580.61 ± 131.96	-0.362	0.718

a *t*’ test.

There were no significant differences between average scores and all of the self-reported joint symptom scores between the two groups, except for the symptom of difficulty going down stairs (Table 2).

**Table 2:** Self-reported joint symptoms scores at baseline

Symptom	Self-reported joint symptoms ( X ± S )		*t*	*P*
	Mega MSM (n=49)	Placebo (n=50)		

Joint pain	3.31 ± 0.68	3.18 ± 0.90	0.786	0.434
Joint stiffness	2.31 ± 1.10	2.46 ± 1.13	-0.686	0.494
Joint swelling	1.78 ± 1.05	1.74 ± 0.94	0.177	0.860
Difficulty walking	2.49 ± 0.87	2.20 ± 1.05	1.495	0.138
Difficulty getting up from bed	2.10 ± 1.07	2.08 ± 0.94	0.109	0.913
Difficulty going down stairs	3.24 ± 0.88	2.78 ± 1.02	2.433	0.017
Average Score	2.54 ± 0.76	2.41 ± 0.67	0.909	0.365

There were no significant differences on joint examination scores and the average score between the two groups, except in the score of skin redness (Table 3).

**Table 3:** Joint examination scores at baseline

Symptom	Joint examination scores ( X ± S )		*t*	*P*
	Mega MSM (n=49)	Placebo (n=50)		

Skin redness	1.31 ± 0.74	1.02 ± 0.14	2.653^a^	0.011
Swelling	1.88 ± 1.11	1.64 ± 0.88	1.183	0.240
Heat	1.43 ± 0.91	1.22 ± 0.51	1.402^a^	0.165
Pain	3.39 ± 0.70	3.18 ± 0.77	1.398	0.165
Joint movement disorder	3.08 ± 0.89	2.92 ± 0.83	0.938	0.351
Average Score	2.22 ± 0.70	2.00 ± 0.45	1.858^a^	0.067

a *t*’ test.

Generally, the two arms balanced well at baseline through randomization.

### Results after intervention

**SF-36 scores after the intervention.** After the 12-week intervention, the score of physiological functioning (PF) in the Mega MSM group was 72.04 ± 17.11, which was significantly higher than the score of 61.60 ± 18.99 in the placebo group (*p*=0.005). The score of MH in the Mega MSM group was 84.33 ± 8.83 and was also significantly higher than the score of 74.88 ± 13.55 in the placebo group (*p*<0.001). Also, the MCS score in Mega MSM group was 324.31 ± 45.30, compared to the score of 304.01 ± 55.50 in the placebo group (*p*=0.049) (Table 4). There was insufficient evidence to distinguish the effects of Mega MSM and the placebo for other aspects.

**Table 4:** SF-36 scores after the intervention

Dimension	SF-36 ( X ± S )		*t*	*P*
	Mega MSM (n=49)	Placebo (n=50)		

PF	72.04 ± 17.11	61.60 ± 18.99	2.872	0.005
RP	54.08 ± 40.94	49.50 ± 50.12	0.499^a^	0.619
BP	70.00 ± 11.37	65.00 ± 13.89	1.962^a^	0.053
GH	58.88 ± 21.66	56.40 ± 18.55	0.612	0.542
VT	73.57 ± 18.65	69.80 ± 16.60	1.063	0.290
SF	71.17 ± 20.12	72.00 ± 18.31	-0.214	0.831
RE	95.24 ± 18.00	87.33 ± 31.51	1.536^a^	0.128
MH	84.33 ± 8.83	74.88 ± 13.55	4.119^a^	0.000
HT	44.39 ± 13.76	44.50 ± 11.62	-0.044	0.965
PCS	255.00 ± 77.43	232.50 ± 86.33	1.366^a^	0.175
MCS	324.31 ± 45.30	304.01 ± 55.50	1.991	0.049
Total score	623.70 ± 122.22	581.01 ± 138.49	1.625	0.107

a *t*’ test.

**Self-reported joint symptom scores after the intervention.** After the intervention, the score of joint pain in the Mega MSM group was 2.51 ± 0.82, lower than the score of 3.04 ± 0.93 in the placebo group (*p*=0.003). The average score in the Mega MSM group was 2.02 ± 0.70, which was also significantly lower than the score of 2.32 ± 0.68 in the placebo group (*p*=0.036) (Table 5). There was insufficient evidence to suggest any differences between Mega MSM and the placebo for other self-reported symptoms.

**Table 5:** Self-reported joint symptoms scores after the intervention

Symptoms	Self-reported joint symptoms ( X ± S )		*t*	*P*
	Mega MSM (n=49)	Placebo (n=50)		

Joint pain	2.51 ± 0.82	3.04 ± 0.93	-3.014	0.003
Joint stiffness	2.00 ± 0.87	2.34 ± 1.08	-1.729^a^	0.087
Joint swelling	1.51 ± 0.82	1.74 ± 0.94	-1.293	0.199
Difficulty walking	1.98 ± 0.80	2.12 ± 1.04	-0.751^a^	0.454
Difficulty getting up from bed	1.76 ± 0.88	2.02 ± 0.92	-1.469	0.145
Difficulty going down stairs	2.37 ± 0.76	2.64 ± 1.05	-1.490^a^	0.140
Average Score	2.02 ± 0.70	2.32 ± 0.68	-2.130	0.036

a *t*’ test.

**Joint examination scores after the intervention.** After the intervention, the score of skin redness in the Mega MSM group was 1.16 ± 0.47 compared to the score of 1.02 ± 0.14 in the placebo group (*p*=0.046), and the score of pain in the Mega MSM group was 2.75 ± 0.76, which was significantly lower than the score of 3.08 ± 0.83 in the placebo group (*p*=0.002). Also, the score of joint movement disorder in the Mega MSM group was 2.37 ± 0.70, lower than that in the placebo group (*p*=0.004) (Table 6).

**Table 6:** Joint examination scores after the intervention

Symptom	Joint examination scores ( X ± S )		*t*	*P*
	Mega MSM (n=49)	Placebo (n=50)		

Skin redness	1.16 ± 0.47	1.02 ± 0.14	2.037^a^	0.046
Swelling	1.49 ± 0.82	1.66 ± 0.87	-1.001	0.320
Heat	1.33 ± 0.75	1.20 ± 0.50	0.992^a^	0.324
Pain	2.75 ± 0.76	3.08 ± 0.83	-3.173	0.002
Joint movement disorder	2.37 ± 0.70	2.84 ± 0.87	-2.987	0.004
Average Score	1.78 ± 0.56	1.96 ± 0.46	-1.700	0.092

a *t*’ test.

**The improvement rates on self-reported joint symptoms, and the joint examination scores before and after the intervention.** Symptom improvement was defined as the scores of self-reported joint symptoms or if the joint examination reduced by 1 point or above. Improvement rates were also calculated and compared between the two groups (Table 7).

**Table 7:** The improvement rates on self-reported joint symptoms and the joint examination scores before and after the intervention

	Symptom	The improvement rate(%)		χ^2^	*P*
		Mega MSM (n=49)	Placebo (n=50)		

1	Joint pain	77.6	16.0	37.691	0.000
2	Joint stiffness	30.6	12.0	5.130	0.024
3	Joint swelling	22.4	0.0	12.628	0.000
4	Difficulty walking	51.0	8.0	22.113	0.000
5	Difficulty getting up from bed	34.7	6.0	12.639	0.000
6	Difficulty going down stairs	77.6	14.0	40.313	0.000
7	Skin redness	8.2	0.0	2.409	0.121
8	Swelling	28.6	0.0	16.639	0.000
9	Heat	10.2	2.0	1.662	0.197
10	Pain	75.5	16.0	35.349	0.000
11	Joint movement disorder	61.2	14.0	23.580	0.000

**The changes of SF-36 scores before and after the intervention.** The changes of PF, RP, BP, SF, PCS, MCS and the total score in the Mega MSM group were larger than those in the placebo group (Table 8).

**Table 8:** The changes of SF-36 scores before and after the intervention (Wilcoxon two-sample rank sum test)

Dimension	Mega MSM (n=49)	Placebo (n=50)	z	*P*
	Average rank	Average rank		

PF	65.41	34.90	-5.878	0.000
RP	59.17	41.01	-4.333	0.000
BP	62.26	37.99	-5.185	0.000
GH	51.53	48.50	-1.181	0.238
VT	51.47	48.56	-1.134	0.257
SF	56.53	43.60	-3.265	0.001
RE	49.50	50.49	-0.557	0.564
MH	51.93	48.11	-1.265	0.206
HT	50.01	49.99	-0.014	0.989
PCS	66.28	34.05	-6.067	0.000
MCS	54.67	45.42	-2.133	0.033
Total score	66.01	34.01	-5.857	0.000

**The rate of adverse events.** The rate of adverse events was 10.2% in the Mega MSM group and 8.0% in the placebo group, respectively, with no significant difference noted (χ^2^=0.001,*p*=0.975). The only side effect reported in the Mega MSM group was stomach discomfort (stomach bloating).

## DISCUSSION

Joint degeneration is a common condition affecting major aging populations. Over long-term administration, current routine therapies, such as non-steroidal anti-inflammatory drugs (NSAIDs), could lead to adverse effects. This clinical trial was designed to determine whether daily administration of Mega MSM, as an alternative, could relieve symptoms in aging populations experiencing joint degeneration.

Mega MSM was found to have positive effects in improving joint function and relieving joint problems associated with joint degeneration–including joint pain, joint stiffness, joint swelling, difficulty walking, difficulty getting up from bed and difficulty going down stairs. It was also found to improve overall quality of life especially in the dimensions PF, RP, BP, SF, PCS, MCS, etc. No serious, adverse reactions from taking Mega MSM were found in the study.

Mega MSM exhibited potent effects in relieving symptoms such as joint stiffness and improving joint functions. The improved rate of nine dimensions were higher in the Mega MSM group than in the placebo group. Overall, Mega MSM had positive effects on relieving joint problems.

This study also showed that participants in the Mega MSM group experienced better quality of life after taking the Mega MSM capsules compared to those taking the placebo. As mentioned in the results section, after a 12-week intervention, the physiological functions score was approximately 10 points greater in the Mega MSM group compared to the placebo group. Also, the mental composite score was 20 points higher than the placebo group. The scores of physiological functions, role-physical, bodily pain and vitality changed in the Mega MSM group after the intervention. The physical composite score and mental composite score were raised, too.

As in many randomized controlled trial studies, we followed ITT policy, ignoring nonadherence. Subjects were compared based on initial randomization intervention groups. This method allowed us to avoid potential biases in comparison based on per-protocol analysis, since there was no evidence to suggest that nonadherence was randomly distributed, though this method might induce the underestimation of the effect size.

There were also several limitations in this study. Some potential confounding factors—such as weight, lifestyle and dietary habits—were not controlled in the study. But as the two groups were well-balanced in the baseline characteristics, we expected that the effects of differences in these, or other potential confounding factors, would be minimal. The results could only be responsible for this study range. When inferences are made to populations different from the study population we specified, there could be a generalizing problem. Further study is needed to confirm these inferences out of the ranges we mentioned. Another limitation was the use of a questionnaire to collect baseline information, which relies on participants’ recall. Thus, the results from this part may be prone to recall bias. Finally, all of our participants were volunteers, i.e. those eligible who did not wish to participate were excluded. If those who were excluded were not exactly compatible with those enrolled for the study, the results would suffer a selection bias.

## CONCLUSION

In summary, Mega MSM shows positive effects in improving joint function, relieving symptoms associated with joint degeneration—including joint pain, joint stiffness, joint swelling, difficulty walking, difficulty getting up from bed and difficulty going down stairs—and improving quality of life, in elderly populations.
